# Supplementary material for: WordCommentsAnalyzer: A windows software tool for qualitative research
Source: F1000Res. 2018 Sep 4;7:536. Originally published 2018 May 3. [Version 2] doi: 10.12688/f1000research.14819.2 (PMC6137411; doi:10.12688/f1000research.14819.2)
Supplement: Supplementary file 3 [file f1000research-7-17614-s0002.tgz › b2a79496-4ada-47c4-9682-a99ce867836d.zip!/Eight Iranian Health Journals/Yafteh _40.docx]

© Yafteh  . This work is licensed under CC BY 4.0 (https://creativecommons.org/licenses/by/4.0/).

Volume & Issue: دوره 11 - شماره 1

Title: بررسی میزان کلیرانس تئوفیلین در بیماران مبتلا به آسم حاد یا بیماری انسدادی مزمن ریوی بستری در اورژانس داخلی بیمارستان افضلی پور کرمان درسال1384

http://yafte.lums.ac.ir/article-1-133-fa.html

تئوفیلین یکی از قدیمی ترین داروهایی است که در درمان آسم و بیماریهای مزمن انسدادی ریه به کار میرود. این دارو توسط آنزیم های کبدی متابولیزه شده و داروهای تغییر دهنده متابولیسم کبدی، کلیرانس آن را تغییر می دهند. عوارض دارو معمولا به غلظت پلاسمایی آن وابستگی داشته و در سطح سرمی بالای mg/L 20 رخ می دهد. این مطالعه به منظور نحوه تجویز آمینوفیلین وریدی و بررسی کلیرانس و سطح سرمی دارو در بیماران بستری در اورژانس داخلی بیمارستان افضلی پور کرمان انجام شد. Ø مواد و روش ها: این مطالعه مقطعی بر روی 21 بیمار مبتلا به COPD و آسماتیک در سال 1384 انجام شد. 2 نمونهء خونی از هر بیمار در فواصل 15 دقیقه و 24 ساعت پس از اتمام دوز بارگیری و شروع انفوزیون نگهدارنده آمینوفیلین گرفته شد. غلظت تئوفیلین سرم توسط دستگاه HPLC اندازه گیری شده و کلیرانس دارو توسط فرمول چو محاسبه و با کلیرانس حاصل از مطالعات فارماکوکینتیک جمعیتی تئوفیلین در افراد غیر ایرانی، مقایسه شد. Ø یافته ها: میانگین سنی بیماران 18 ± 57 سال بود. میانگین غلظت تئوفیلین در زمانهای ذکر شده به ترتیب عبارت از 4 ± 1/9 و mg/L 3/3 ± 1/5 بود. کلیرانس تئوفیلین با استفاده از فرمول چو در بیماران ما L/hr 21/2 ± 2/4 به دست آمد که با کلیرانس حاصل از مطالعات جمعیتی (1 ± 1/3) تفاوت معنی داری نداشت (075/0 p= ). بحث و نتیجه گیری: نحوه تجویز آمینوفیلین تزریقی در بیماران ما اصلا مناسب نبوده به طوریکه در 62% بیماران، سطح خونی تئوفیلین کمتر از mg/L 5 و زیر حداقل سطح درمانی بود. پیشنهاد می شود در بیمارانی که تئوفیلین و به خصوص فرم تزریقی آنرا دریافت می کنند برای اطمینان از قرار داشتن سطح سرمی در محدوده درمانی، اثر بخشی لازم و نیز جلوگیری از بروز عوارض ناخواسته دارویی، مانیتورینگ سطح سرمی انجام شود.

Title: میزان موفقیت ترک اعتیاد به روش سم زدایی فوق سریع (UROD) بیهوشی عمومی

http://yafte.lums.ac.ir/article-1-134-fa.html

Ø مقدمه: اعتیاد به مواد مخدر علاوه بر زیانهای جسمی، تبعات اجتماعی، اقتصادی بسیاری دارد. روشهای مختلفی برای ترک اعتیاد استفاده می شود، یکی از روشهای جدید سم زدایی فوق سریع (UROD) است که منجر به کاهش علائم و عوارض سندرم ترک می شود. Ø مواد و روش ها: این مطالعه مقطعی با هدف تعیین میزان موفقیت ترک اعتیاد به روش UROD در 112 بیمار مراجعه کننده به بیمارستان شهدای عشایر خرم آباد در سال 1384 انجام گردید. بیماران پس از انجام مصاحبه و اخذ رضایت نامه به مدت 4 تا 5 ساعت و با نالوکسان با دوز mg/kg 07/0-05/0 به صورت انفوزیون وریدی تحت بیهوشی عمومی قرار گرفتند. ارزیابی درمان به صورت ویزیت ماهیانه و سه نوبت تست اعتیاد به صورت تصادفی در مدت 4 تا 6 ماه صورت گرفت. در این مدت همه بیماران تحت نظر روان پزشک قرار گرفتند. داده ها توسط نرم افزار SPSS ورژن 5/11 و با استفاده از آزمون آماری x2 مورد تجزیه و تحلیل آماری قرار گرفتند. Ø یافته ها: از 112 بیمار تحت درمان، 104 نفر در پیگیری شش ماهه شرکت داشتند، میزان موفقیت ترک اعتیاد در این روش پس از گذشت این زمان 5/86 درصد برآورد گردید. در این مطالعه بین متغیرهای سطح تحصیلات، سن، نوع ماده مصرفی، مدت زمان مصرف و میزان موفقیت رابطه معنی داری به دست نیامد. ولی در خصوص وضعیت اشتغال با موفقیت ترک این رابطه معنی دار بود (001/0 p< ). بحث و نتیجه گیری: در مطالعات مختلف میزان عود پس از 6 ماه بین 25 تا 68 درصد گزارش گردیده است. در حالیکه در این مطالعه 5/86 درصد پس از 6 ماه وابستگی به مواد مخدر نداشتند. بنابراین UROD به عنوان یک روش کم عارضه درمان برای قطع وابستگی همراه با روشهای درمانی تکمیلی ضروری به نظر می رسد

Title: بررسی مقایسه ای اثر ژل گیاه صبر زرد (Aloe vera) روی درمان زخمهای مزمن در مقایسه با درمانهای رایج

http://yafte.lums.ac.ir/article-1-135-fa.html

مقدمه: امروزه درمان زخم های مزمن و درمان آنها یکی ازپر هزینه ترین مسائل حل نشده سلامتی هستند. در حال حاضر روشهای درمان زخم های مزمن شامل تمیزکردن زخم، درمان آنتی بیوتیکی و گاهیً جدا کردن بافتهای اسیب دیده با روش جراحی است که در مواردی مثل زخم دیابتی و زخم بستر سریعترین و موثرترین روش است. هدف از این مطالعه بررسی مقایسه اثر ژل گیاه صبر زرد بر کیفیت بهبود و کاهش مدت زمان بهبودی زخم مزمن است. مواد و روش ها: تعداد 30 نفر از افرادی که قبلاً تحت درمانهای رایج قرار گرفته بودند اما پاسخ درمانی ندیده بودند از بین مراجعین به کلینیک های مختلف یا بیمارستان به روش غیر تصادفی انتخاب شدند و سپس به درمان روزانه 2 تا 3 بار ژل صبر زرد 2% به فرم موضعی بر روی زخمهای مزمن پرداخته شد. با توجه به استاندارد زمانی برای درمان زخم های نارسایی عروقی حداکثر 3 ماه برای و برای درمان زخم بستر حداکثر یک ماه در نظر گرفته شد وهر 2 هفته یکبار زخمها ویزیت شدند. سپس اطلاعات مربوط به بیماران با مشاهده و تکمیل پرسشنامه جمع آوری شد. نوع مطالعه آینده نگر و مقطعی بود. گروه تجربی قبل از شروع درمان با صبر زرد به عنوان گروه شاهد نیز در نظر گرفته شد و به مطالعه زوجی یبن خود موارد پرداخته شد. یافته ها: از بین 30 مورد، 27 مورد (90%) پاسخ کامل به درمان و10% عدم پاسخ داشتند. متوسط زمانی مورد نیاز برای پاسخ کامل به درمان 25/31 روز بود که حداکثر زمان مربوط به بهبود زخم بستر با زمان حداکثر 90 روز و متوسط زمان 1/58 روز برای زخمهای بستر و کمترین زمان بهبودی مربوط به زخمهای جراحی با زمان حداقل 3 روز و متوسط زمانی 11 روز بود. در بررسی فراوانی نوع بهبود نهایی زخم با درمان سرپائی و بستری، سن، جنس، وجود یا عدم وجود بیماری زمینه ای، عمق و سطح زخم تفاوت معنی داری مشاهده نشد اما تفاوت معنی داری در مدت زمان لازم برای کاهش قرمزی، تورم، ترشح و ترمیم کامل زخم مشاهده شد. Ø بحث و نتیجه گیری : در کل چنین به نظر می رسد که ژل گیاه صبر زرد در کیفیت و سرعت بهبود زخم نسبت به درمان رایج بسیار موثرتر و کم هزینه تر است.

Title: بررسی مقایسه ای آگاهی و نگرش دانش آموزان مقطع متوسطه نسبت به اکستازی در مناطق آسیب خیز و غیرآسیب خیز شهر خرم آباد در سال 1385

http://yafte.lums.ac.ir/article-1-136-fa.html

مقدمه: ارزیابی های صورت گرفته نشان دهنده شیوع چشمگیر سوء مصرف اکستازی به عنوان ماده مخدر مورد علاقه جوانان درسالهای اخیر بوده است. مطالعه حاضر به منظور بررسی میزان آگاهی و نگرش دانش آموزان مقطع متوسطه نسبت به اکستازی در مناطق آسیب خیز و غیرآسیب خیز شهر خرم اباد انجام گرفته است. مواد و روش ها: در این مطالعه مقطعی 200 نفر از دانش آموزان به روش تصادفی چند مرحله ای در هر دو جنس دختر و پسر انتخاب و مورد بررسی قرار گرفتند. ابزار گردآوری اطلاعات، پرسشنامه ای شامل 50 سوال بود. اطلاعات با استفاده از روش های آماری از جمله آنالیز واریانس مورد تجزیه و تحلیل قرار گرفت. یافته ها: نتایج نشان داد در مناطق آسیب خیز شهر 2/6 درصد از دانش آموزان نگرش مثبت و4/45 درصد نگرش بسیارمنفی داشتند و در مناطق غیرآسیب خیز 2 درصد دانش آموزان نگرش مثبت و 4/58 درصد نگرش بسیار منفی به ماده اکستازی داشتند. در بررسی سطوح آگاهی، 29/44 درصد مناطق آسیب خیز آگاهی ضعیف، و 1/7 درصد دانش آموزان از آگاهی بالایی برخوردار بودند. در مناطق غیر آسیب خیز5/48 درصد آگاهی ضعیف، 5/42 درصد آگاهی متوسط و 5 درصد آگاهی بالایی نسبت به این ماده داشتند.آزمون های آماری نشان داد هیچ ارتباط معنی داری بین سطوح آگاهی و نگرش دانش آموزان با تحصیلات والدین، شغل والدین، جنس، سن و تعداد فرزندان وجود ندارد. ارتباط معنی داری بین سطوح آگاهی دانش آموزان با رتبه تولد و معدل دانش آموزان مشاهده شد در حالی که این دو متغیر با میزان نگرش ارتباط معنی داری نداشتند. بحث و نتیجه گیری : با توجه به یافته های تحقیق پیشنهاد می گردد افزایش آگاهی عمومی از طریق رسانه های اجتماعی صورت گیرد که لزوم انجام این برنامه ها در مناطق آسیب خیز بیشتر می باشد.

Title: افزودن امگا 3 به درمان نگهدارنده در افسردگی راجعه تک قطبی

http://yafte.lums.ac.ir/article-1-137-fa.html

مقدمه: افسردگی یکی از شایعترین اختالات روانپزشکی است. بسیاری از بیمارانی که با تشخیص افسردگی تحت درمان دارویی قرار می گیرند علی رغم مصرف داروهای ضدافسردگی، اپی زود های تکرار شونده افسردگی را دارند. مطالعات بسیاری میزان پایین افسردگی را درکشور هایی که مصرف غذاهای دریایی دارند نشان داده اند. مواد و روش ها: مطالعه حاضر با هدف بررسی تاثیر امگا3 به عنوان درمان مکمل در بیماران با افسردگی راجعه تحت درمان نگهدارنده با داروهای ضد افسردگی انجام گرفته است.مواد وروشها:در یک کارازمایی بالینی 80 بیمار با تشخیص افسردگی در دو گروه 40 نفری در مطالعه شرکت کردند. در یک گروه درمانهای استاندارد ضدافسردگی (با داروهای SSRI ) ودر گروه دوم امگا 3 به عنوان درمان مکمل به درمان افسردگی (با داروهای SSRI ) اضافه شد. Ø یافته ها: یافته های تحقیق نشان داد امگا 3 بطور چشمگیری موجب کاهش نمره افسردگی بیماران تحت درمان با این دارو و افزایش عملکرد آنها گردید. Ø بحث و نتیجه گیری : با توجه به یافته های تحقیق به نظر می رسد امگا 3 می تواند به عنوان مکمل در کنار داروهای ضد افسردگی جهت بیماران با افسردگی های راجعه و مقاوم به درمان داروئی به کار رود.

Title: تولید مونوکلونال آنتی بادی علیه پروتئین سطحی شماره 2 مروزوئیت( MSP-2) و بررسی ایمنی زایی دومن های مختلف این مولکول

http://yafte.lums.ac.ir/article-1-852-fa.html

مقدمه: مولکول سطحی شماره 2 مروزئیت (MSP-2)پلاسمودیوم فالسیپاروم یکی از کاندیداهای واکسن علیه مالاریا است و نتایج مطالعات مختلف بیانگر ایمنی زا بودن قسمت متغیر این مولکول است. مونوکلونال آنتی بادی علیه MSP-2 روشی مطمئن و قطعی برای تایید این ایمنی زایی است. مواد و روش ها: انگل پلاسمودیوم فالسیپاروم در محیط کشت حاوی RPMI 1640 کشت داده شد و آنتی ژنهای شیزونت بصورت خام استخراج و به موش تزریق و طحال موش خارج گردید. لنفوسیت های B استخراج شده و فیوژن با سلول های NS-1 صورت گرفته و مونوکلونال آنتی بادی علیه MSP-2 تولید گردید. بحث و نتیجه گیری: تولید مونوکلونال آنتی بادی علیه آنتی ژنهای سطحی مروزوئیت در پلاسمودیوم فالسیپاروم مانند سایر آنتی ژنها یکی از روشهای قابل اعتماد بخصوص در ارزیابی کیفی آنتی ژن و نیز در تایید ایمنی زایی آن است و در مطالعه حاضر این روش به درستی آنتی ژن MSP-2 را مورد ارزیابی قرار داد. یافته ها: تعدادی مونوکلونال آنتی بادی علیه MSP-2 ساخته شده همگی تائید کننده ایمنی زایی بلوک های متغیر مولکول MSP-2 بوده و نتایج آزمایشات بررسی ایمنی زایی مولکول MSP-2، با استفاده از سرم بیماران مبتلا به مالاریا را تائید نمود.

Title: بررسـی استانداردهای کیف های کودکان دبستانی شهر خرم آباد سا ل تحصیلی 87- 1386

http://yafte.lums.ac.ir/article-1-138-fa.html

مقدمه: بسیاری از دانش آموزان تمایل به حمل کیف های کوله پشتی دارند. حمل کیف های سنگین بطور فزاینده دانش آموزان را در معرض خطر قرار داده و ممکنست منجر به آسیب های دراز مدت به بدن در حال رشد آنها شود. این تحقیق بر روی دانش آموزان دبستانی شهر خرم آباد جهت بررسی وزن کیف های آنها و نحوه بارگیری و حمل کیف ها ی آنها ، نحوه بار گیری و حمل کیف ها و وجود سابقه کمر درد در آنها انجام شده است . مواد و روش ها: این مطالعه توصیفی _ مقطعی روی 1009 دانش آموز دبستانی انجام شد. دانش آموزان و کیف های آنها توزین شده و درصد وزن کیف از وزن دانش آموز تعیین شد. در عین حال سوالاتی در مورد نحوه بار گیری ، حمل کیف ها و سابقه کمر درد در کودکان انجام و ثبت گردید . یافته ها: 8/57% از کودکان مورد بررسی کیف های سنگین حمل می کردند. 6/24% تنها از یکی از بند های شانه برای حمل کیف استفاده می کردند و سابقه کمر درد در 1/26% آنها وجود داشت. رابطه معنی داری بین کیف های سنگین و حمل آنها روی یک شانه و سابقه کمر درد در کودکان وجود داشت . بحث و نتیجه گیری : بسیاری از دانش آموزان کیف های سنگین تر از وزن استاندارد و با روش غلط حمل می کنند که منجر به ایجاد کمر درد و سایر عوارض در آنها می شود .

Title: بررسی میزان ابتلا بهHIV و HCV دربیماران مشکوک مراجعه کننده به بیمارستان شهدای عشایر خرم آباد در سال 1386

http://yafte.lums.ac.ir/article-1-139-fa.html

مقدمه: شیوع HIV و HCV در کشورهای مختلف با توجه به ریسک فاکتورهای اختصاصی و دیگر فاکتورها متفاوت است. به ویژه زمانی که عفونت همزمان وجود داشته باشد که این خود می تواند تاثیرات خاصی را روی روند عفونت، بقا و نیز میزان شیوع آن ها داشته باشد. هدف از این مطالعه بررسی میزان شیوع این عفونت ها به خصوص عفونت همزمان می باشد. مواد و روش ها: این مطالعه یک مطالعه توصیفی- مقطعی می باشد که در سال 1386 بر روی مراجعین مشکوک به بیماری های HIV و HCV ‌ انجام گرفت که از نظر وجود آنتی بادی با استفاده از آزمون الایزا مورد بررسی قرار گرفتند و پس از جمع آوری داده ها و وارد کردن آنها در نرم افزار آماری SPSS نتایج مورد تجزیه و تحلیل قرار گرفتند. از آزمون دقیق فیشر و با در نظر گرفتن سطح خطای 5 ٪ برای تعیین ارتباط بین میزان ابتلا به بیماری ها و جنس استفاده گردید. یافته ها: دراین مطالعه 304 نفر مرد و116 نفر زن بودند. نتایج این مطالعه نشان داد که تعداد موارد HIV مثبت 45 نفر 7/10٪ (10 ٪ مرد، 7/0 ٪ زن) و HCV مثبت 115 نفر 8/24٪ (3/20% مرد و 5/4% زن) بودند.میزان ابتلا به عفونت همزمان HCV+/HIV+) ) درکل افراد جامعه مورد مطالعه 1/8٪ بودند. که در این میان 6/75% افرادی که HIV مثبت بودند نیز HCV مثبت بودند.همچنین بین میزان ابتلا به HIV و HCV ارتباط معنی داری مشاهده گردید(001/0 (p< . بحث و نتیجه گیری : با توجه به بالا بودن میزان ابتلا به HCV در افرادی که HIV مثبت هستند و ارتباط معنا داری که وجود دارد به نظر می رسد افرادی که HIV مثبت هستند بیشترین احتمال و خطر را برای کسب و یا انتقال HCV را داشته و دارند. در ضمن تفاوت قابل ملاحظه ای بین میزان ابتلا به عفونت همزمان در کشور ما با دیگر کشور ها وجود دارد.

Title: بررسی آلودگی کلی فرم در فرایند تولید یخ کارخانجات یخ سازی شهرستان کرمان

http://yafte.lums.ac.ir/article-1-140-fa.html

Ø مقدمه: اهمیت انتقال عوامل بیماریزا از طریق مصرف یخ کمتر از اهمیت انتقال آن از طریق آب نمی باشد. آلودگی یخ در اثر استفاده از آب آلوده، حمل و نقل، توزیع بهداشتی و نحوه نامناسب نگهداری آن ایجاد می شود. هدف از انجام این مطالعه ارتقاء سطح بهداشت عمومی جامعه از طریق شناخت و کنترل آلودگی میکروبی انواع کلیفرم ، در فرایند تولید یخ در کارخانجات یخ سازی شهرستان کرمان می باشد. Ø مواد و روش ها: مطالعه مقطعی- توصیفی و روش جمع آوری داده ها سرشماری است. که طی آن وضعیت کل باکتریهای کلیفرم در چهار کارخانه یخ سازی در شهرستان کرمان بررسی شده است. نمونه های آب و یخ از سه نقطه آب ورودی، فرایند، یخ خروجی هر کارخانه برداشت گردید. نمونه برداری در نیمه اول سال 1386 انجام گرفت .نمونه ها در دو ماهه اول و سوم فصول بهار و تابستان سال 86 و در هر ماه ده بار برداشت شد. تعداد کل نمونه ها در مدت بررسی 120 نمونه می باشد. برای مقایسه داده ها از آزمون t-test و x2 و نرم افزار SPSS استفاده شد. Ø یافته ها: نتایج نشان داد که از 120 نمونه جمع آوری شده جمعاً 27 نمونه آلودگی کلیفرم داشته اند. کمترین موارد آلودگی در آب ورودی 5/7%، و بیشترین آلودگی مربوط به یخ خروجی 5/37 % بود. کلیه کارخانجات آلودگی کلیفرم داشته اند. کمترین آلودگی مربوط به کارخانه شماره 1، 18/18% و بیشترین آلودگی مربوط به کارخانجات شماره 2 و4 ، 25% برای هر کدام می باشد. بررسی انجام شده نشان داد که علیرغم عدم آلودگی آب ورودی، یخ تولیدی دارای آلودگی است Ø بحث و نتیجه گیری : این نتایج حاکی از آن است که آلودگی محیط کارخانه و آلودگی کارکنان به اندازه تصفیه ناکافی آب می تواند در انتشار باکتریها و بیماریهایی منتقله از آب نقش داشته باشد. توصیه می شود که در کلیه کارخانجات با ارتقاء سطح بهداشت محیط، کنترل فرایند تولید و همچنین رسیدگی به وضعیت بهداشت فردی کارکنان درجه آلودگی را تقلیل و رفع نمود. ضمنا در مورد بهداشتی نمودن مراحل حمل و تولید یخ مراقبت بیشتری معمول گردد.

Title: بررسی مقدار فلزات سنگین کادمیوم، سرب، کروم و نیکل در ماهیان پرورشی شهرستان خرم آباد در بهار و تابستان سال 1385

http://yafte.lums.ac.ir/article-1-141-fa.html

مقدمه: استفاده از منابع خوراکی آبزی به ویژه ماهیان به عنوان بخشی از منابع پروتئینی ، به دلیل نیاز روز افزون انسان به غذا، در حال افزایش است. مطابق آمارهای موجود، مصرف سرانه آبزیان در جهان از 3/14 کیلوگرم در سال 1994 به بیش از 75/15 کیلوگرم در سال 1997 افزایش یافته است. مصرف آبزیان در ایران از کمتر از 1 کیلوگرم در سال 1357 به 57/4 کیلوگرم در سال 1375 افزایش یافته است. بنابراین به موازات افزایش مصرف این منابع، اهمیّت بهداشتی و سلامتی ماهیان نیز، بیشتر می گردد که در این میان فلزات سنگین به علت فرایند بیولوژیکی و تجمع زیستی، تشخیص و اندازه گیری اهمیت زیادی دارند. در این مطالعه توصیفی مقطعی مقدار فلزات سنگین کادمیوم، سرب، کروم و نیکل در ماهیان پرورشی شهرستان خرم آباد در بهار و تابستان سال 1385 مورد بررسی قرار گرفته است . Ø مواد و روش‌ها: به منظور بررسی غلظت فلزات سنگین کادمیوم، کروم، سرب و نیکل ماهیان پرورشی شهر ستان خرم آباد بطور منظم در فواصل زمانی مشخص در طی فصل بهار و تابستان تعداد 240 نمونه از ده محل پرورش ماهی جمع آوری گردید و مقدار فلزات سنگین کادمیوم، کروم، سرب و نیکل موجود در بافت ماهیان پس از عملیات هضم شیمیایی توسط دستگاه جذب اتمی تعیین شد. و نتایج با استفاده از نرم افزار SPSS و دو آزمون T - Test ت جزیه و تحلیل گردید. در آزمایشهای از مواد شیمیایی با درجه خلوص بسیار زیاد استفاده گردید . Ø یافته‌ها: میانگین غلظت سرب، کروم، کادمیوم ونیکل در بافت ماهیان مورد مطالعه به ترتیب برای محلهای مورد نظر 46/0، 06/0، 053/0 و3/0 میلی گرم بر لیتر بر حسب وزن خشک ماهی بدست آمد. نتایج حاصل از انجام این پژوهش نشان داد که مقدار فلزات سنگین سرب، کادمیوم، کروم و نیکل به ترتیب در 27%، 8%، 3% و 25% نمونه های مورد مطالعه از حداکثر مجاز سازمان بهداشت جهانی کمتر بود . Ø بحث و نتیجه‌گیری: نتایج نشان داد که توزیع فلزات سنگین در بافت ماهیان مورد مطالعه بطور تقریباً یکسانی وجود دارد و از نظر آماری اختلاف معنی داری بین غلظت آنها مشاهده نگردید ولی مقادیر این فلزات در ماهیان پرورشی بیشتر از ماهیان دریایی بود. مقایسه مقادیر بدست آمده با استاندارد سازمان بهداشت جهانی نشان داد که مقدار فلزات سنگین سرب، کروم، کادمیوم، و نیکل از حد مجاز سازمان بهداشت جهانی کمتر است .

Title: گزارش یک مورد تومور نادر مجاری هوایی مقلد آسم به نام شوانوما

http://yafte.lums.ac.ir/article-1-142-fa.html

مقدمه: شوانومای اولیه تراشه و برونش بسیار نادر می باشد و می تواند با انسداد راه هوایی سبب ایجاد علائم مشابه آسم گردد. معرفی مورد : خانم 17 ساله ای را معرفی می نماییم که با شوانومای برونش چپ به مدت یک سال با تشخیص اولیه آسم و آلرژی تحت درمان بود که با بررسی برونکوسکوپی تومور پایه دار برونش چپ مشخص گردید که در طی دم و باز دم از دهانه برونش بیرون می زد و باعث بسته شدن بیش از 80% داخل مجاری شده بود که تحت رزکسیون و برونکوپلاستی حفظ ریه قرار گرفت و تمامی علائم بیمار از بین رفت بحث و نتیجه گیری: پس باید دقت کنیم که تومورها حتی این تومور نادر نیز بایستی در تشخیص افتراقی بیمارانی که با علائم انسداد راه هوایی مراجعه می نمایند به خصوص در کودکان و جوانان وقتی به درمانهای معمول جواب نمی دهند مد نظر قرار گیرد.
